# The Role of Parental Health Literacy in Establishing Health-Promoting Habits in Early Childhood

**DOI:** 10.3390/children11050576

**Published:** 2024-05-10

**Authors:** Melinda Csima, Judit Podráczky, Viktória Keresztes, Evelin Soós, Judit Fináncz

**Affiliations:** 1 Institute of Education, Hungarian University of Agriculture and Life Sciences, H-7400 Kaposvár, Hungary; podraczky.judit@uni-mate.hu (J.P.); financz.judit@uni-mate.hu (J.F.); 2 MTA-MATE Early Childhood Research Group, H-7400 Kaposvár, Hungary; keresztes.viktoria98@gmail.com (V.K.); sooos.evelin@gmail.com (E.S.); 3 Education and Society Doctoral School of Education, University of Pécs, H-7624 Pécs, Hungary

**Keywords:** health literacy, health-promoting-habits, health behavior, early childhood, preschool children, parents

## Abstract

In early childhood, children are extremely susceptible to the acquisition of habits and the establishment of health-promoting habits. Therefore, the patterns, routines, and rules transmitted and expected by the adults surrounding the child are of paramount importance and can correlate with the level of their health literacy. Our cross-sectional, quantitative, exploratory study aimed to examine the relationships between parental health literacy and preschool children’s health-related habits, using simple, non-random sampling (*n* = 598). In addition to the sociodemographic characteristics, the measuring tool we compiled included the standardized European Health Literacy Survey Questionnaire (HLS-EU-Q16), as well as a set of questions containing 30 statements suitable for exploring children’s habit systems. The health literacy of the parents involved in our study proved to be more favorable than that of the general population. Regarding children’s habit systems, we found significant differences in several areas by age group (*p* < 0.05) and gender (*p* < 0.05). The levels of parental health literacy (0.003 ≤ *p* ≤ 0.048) and parents’ education (*p* < 0.05) show a correlation with the children’s health-related habit systems: the indicators of children with parents who have a higher level of health literacy and a higher level of education are more favorable in terms of established habits. In the long term, the formation of health-promoting habits may facilitate the internalization of favorable health behavior motives for the future, contributing to the establishment of positive physical, mental, and social health in adulthood.

## 1. Introduction

Several attempts have been made to define and measure health literacy since this term first emerged in the 1970s [1]. Its interpretation, initially approached from a biomedical perspective, has become increasingly multidisciplinary [2]. The contemporary definition of health literacy considers the extent to which individuals can gain, process, and understand fundamental health information to inform optimal decisions concerning their health [3,4]. Several tools for measuring health literacy have been developed over the past few decades [5,6,7], with the most widely used tool currently being the 47-item European Health Literacy Survey Questionnaire [8] or its abbreviated counterpart [9,10,11,12]. Research on the health literacy of specific populations [13,14,15] and on specific health literacy domains [16,17,18] has become increasingly common. In relation to early childhood, Bánfai et al. (2022) [19] pointed out in their systematic review that studies related to health literacy are less focused on children themselves, but rather on the parents and educators raising them. During the examination of parental health literacy, the majority of researchers focused on a specific area which provides information on health-promoting habits and on knowledge and skills related to some disease or condition that often affects children [20,21,22,23,24,25,26]. Based on the conclusions of the studies, enhancing parental health literacy has a positive effect on the health status and health behavior of children, which, in the long term, can contribute to the reduction in health inequalities regarding deprived social groups.

There have been several attempts worldwide to precisely define the term “habit” [27,28,29,30]; however, as it is a rather complex concept, it is not easy to find a general definition for it [31]. Summarizing the different approaches, a habit can be considered as a process during which a stimulus automatically triggers an action impulse owing to previously experienced stimulus–response associations [31,32]. The formation of habits takes place in four stages; a habit is formed when a person (1) decides on a particular action, (2) behaves based on their decision, (3) repeats it (this is the critical phase where maintaining motivation may require support), and (4) in a way that helps establish habit associations [32,33,34,35]. The results of studies based on self-reports [34,35] have highlighted that in the initial stage of the habit formation process, it is important for the individual to have appropriate support, which helps them to maintain motivation and thus make the new habit automatic [30,32]. In terms of habit formation, the early years of life are crucial [36,37]. In families where habits are part of everyday life, children are more cooperative with their parents, and their social competencies are more developed [38]. As a result of the increasingly emphasis on a healthy lifestyle, more and more research is examining the ways of forming health-related habits [32,34,35,39]; however, there are limited measuring tools specifically tailored to young children [19,40]. 

In recent years, several studies have aimed to understand parental health literacy [20,21,22,23,24,25,41] and health-supporting habits formed in early childhood [40,42,43]; however, the connection between these two areas has not been explored until now. Research on the relationship between health literacy and health behavior has primarily been carried out among adolescents [44,45]. In the case of preschool children, studies mainly focus on health-promoting habits [46], since at this age health behavior is established through habits and daily routines. When exploring the relationship between parental health literacy and health-promoting habits of their preschool children, research has typically focused on a special area, such as sleep [47,48], nutrition [49,50,51], or oral hygiene [52]. A complex, comprehensive study of general parental health literacy and children’s health-promoting habits has not been carried out so far; therefore, our study can be considered as filling a gap and can encourage interdisciplinary research.

Accordingly, the aim of our study was to examine the correlations between parental health literacy and the health-promoting habits of young children. In our research, we sought answers to the following questions:

- What characteristics describe the health literacy of parents raising preschool-age children?
- What factors differentiate the health literacy of parents included in the study?
- What habits do preschool children have in relation to a healthy lifestyle?
- What relationship can be found between parental health literacy and the health-related habits and daily routines of young children?

## 2. Methods

### 2.1. Procedure and Measures

To answer our research questions, we conducted a cross-sectional, quantitative, exploratory study among parents raising 3–7-year-old preschool children (*n* = 598) in January 2024, in South-West Hungary. The paper-and-pencil survey was conducted by personal inquiry, with the assistance of interviewers.

In addition to the questions on sociodemographic characteristics, the Hungarian version of HLS-EU-Q16 was incorporated in the measuring tool used [10,53]. To explore children’s habit systems, we compiled a self-designed questionnaire consisting of 30 items. With this questionnaire, we collected information about habits related to diet, hygiene, accident prevention, physical activity, and daily routines. 

The Health Literacy EU-Q16 questionnaire is the shortened version of the 47-item HLS-EU-Q47, which is used to measure health literacy with 16 questions. Following the evaluation guidelines [8,54] for the HLS-EU-Q47 questionnaire, we computed the overall health literacy index, and formed three additional, well-distinguishable sub-indices, named according to the terminology used in previous research. During the creation of the indices, the data of parents who gave more than two incomplete responses were excluded from the analysis. For both the overall health literacy index and the sub-indices, Cronbach’s Alpha was calculated to ensure the reliability of the applied measuring tool (Table 1). Similar to previous studies [10,11,12], both the entire questionnaire and its subscales showed high internal reliability and were therefore considered suitable for analysis.

To see how aware parents were of the recommendations for particular health-promoting habits, we asked them in the form of open-ended questions about the age at which regular toothbrushing is recommended according to their knowledge, as well as how much physical activity 4-year-old children need per day and how much time they can spend in front of a screen. 

To understand the children’s habit systems, we compiled a series of items consisting of 30 statements. During the compilation of the measuring tool, we addressed the issues that receive greater emphasis in the process of early childhood habit formation. Thus, we focused on habits related to nutrition, hygiene, and physical activity, as well as habits related to the daily routine. The individual statements provide information both on the presence of health-promoting habits and on daily routines, for which parents were offered four response options, “never”, “rarely”, “often”, and “always”, characteristic of the preschool child. With the exception of two statements (27 and 28), the response options “often” or “always” indicate that the health-supporting habits are firmly embedded in the young child’s habit system. 

During the statistical analysis, descriptive statistics were used to characterize participant responses on sociodemographic questions, HLS-EU-Q16, and children’s habits. The Chi-Square Test and Fisher’s Exact Test were used to examine the associations between the variables (parental health literacy and education level with respect to children’s age, gender, and habits), as applicable. Depending on the result of the Chi-Square Test, we used the Tukey test to determine which attributes showed significant differences. For measuring effect size, Cramer’s V or Phi coefficients were applied. After the bivariate analyses, multivariate analyses were conducted using binary logistic regression for those habits as a binary outcome variable where at least two explanatory variables—parental health literacy level, parental education level, age, or gender of the child—proved to be significant. Data recording and statistical analyses were performed using IBM SPSS Statistics 29.0.

### 2.2. Sampling and Participant

The required sample size and statistical power were determined using the G*Power 3.1.9.7 software. The analysis calculated a minimum total sample size of 430 participants, with an effect size of 0.2, a significance level (α error prob.) of 0.05, and a statistical power (1 − β error prob.) of 0.95. Considering the minimum sample size required, we approached 650 parents through interviewers, of which 598 participated in the study. During the survey, convenience sampling was used. Respondents gave their consent to participate in the research, their anonymity was ensured, and the research data were kept confidential. We aimed to include a similar proportion of parents with different levels of education in the sample. 

Mostly, the children’s mothers were included in our study (*n* = 513, 85.8%), and the proportion of fathers filling in the questionnaire was below 15%. The average age of parents raising preschool children was 36.44 years (SD = 6.013; Min = 21; Max = 54). The respondents were classified into three groups according to their education level: persons with (1) lower secondary education, (2) upper secondary or post-secondary education, or (3) tertiary education, of at least Bachelor level (Table 2). From the perspective of health literacy, it is particularly important to question whether the respondent has healthcare qualifications, as possessing such qualifications undoubtedly results in a higher level of knowledge regarding health and health preservation. Only 10.5% (*n* = 63) of the examined parents could be classified in this category. 

For items related to habits embedded in our measuring tool, we asked parents to assess, for the youngest of their preschool-aged children, how regularly the child performs the particular activity. The gender distribution of children represents the composition of the population (girls: 50.7%; boys: 49.3%). In accordance with Hungarian regulations, the age of preschool children was between three and seven years, with an average of 61.11 months (SD = 11.75; Min = 33; Max = 81). During the analyses, the children were categorized into four groups based on their age: (1) under 4 years (*n* = 96; 16.1%); (2) between 4 and 5 years (*n* = 198; 33.1%); (3) between 5 and 6 years (*n* = 196; 32.8%); or (4) over 6 years (*n* = 108; 18.1%).

## 3. Results

### 3.1. Health Literacy Characteristics of the Parents Included in the Study

The answers to the questions of the HLS-EU-Q16 questionnaire were dichotomized, following the recommendations of the HLS-EU research group [55]: “easy” (“fairly” or “very” easy = 1) and “difficult” (“fairly” or “very” difficult = 0). In Table 3, the absolute and relative frequencies of the responses to each item by sub-index are displayed. For each of the three sub-indices, there are questions for which the responses of the parents reflect a more difficult navigation in health literacy, and there are questions that show the respondents’ higher level of awareness. In the case of the “Healthcare system-related competence” sub-index, the data show that the utmost challenge lies in judging when it may be necessary to request an additional medical opinion, and the least problematic is to understand the doctor’s or pharmacist’s instructions. In the case of the “Prevention” sub-index, understanding the necessity of screening tests is the least troublesome, while it is clear that respondents are more uncertain about information from the media. The difficulty in understanding information from the media is also reflected in the “Health promotion” sub-index.

The score of the scale, which takes a value between 0 and 16, was calculated as the sum of the scores given to the individual items, with a higher score indicating a higher level of health literacy. In the same way as described in previous studies [9,10,11,12], based on the score achieved on the scale, the respondents were categorized into three groups: groups of parents with inadequate (0–8 points: *n* = 80; 13.4%), problematic (9–12 points; *n* = 183; 30.6%), and sufficient (13–16 points; *n* = 335; 56.0%) health literacy.

Parents’ knowledge of health-promoting habits was assessed in the form of open-ended questions. Responses to the question of toothbrushing were grouped into four categories. One group included responses that linked toothbrushing to age; some people would brush their baby’s teeth from birth, some people indicated infancy, and some respondents named the age of two as the beginning of toothbrushing. The next group included answers that consider toothbrushing necessary from the appearance of the first teeth. In the third group were the answers according to which it is recommended to start toothbrushing at the beginning of complementary feeding, while according to the fourth group it depends on the individual development of the child. The relevant professional recommendations [56] advocate starting regular toothbrushing from the appearance of the first baby teeth so that it can be more easily integrated into the child’s developing habit system. 

Regarding sleep, more than three-quarters of the respondents consider 10–12 h of sleep per day as necessary for a 4-year-old child, which roughly corresponds to the WHO recommendation of 10–13 h [56]. In terms of physical activity/exercise, the parents’ opinions were divided: according to 40.4% of the respondents, 0–60 min of physical activity per day is necessary, another 32.8% said that 61–120 min would be ideal, while 26.8% thought that a 4-year-old child should spend at least 120 min in physical activity. Less than half of the latter group (11.1% of the responding parents) indicated at least 180 min of exercise as recommended by WHO [56]. The parents’ opinions were similarly divided regarding the amount of time spent in front of the screen. Almost 40 percent (39.3%) of the respondents set the maximum amount of daily screen use as less than 1 h (which corresponds to the WHO recommendation [56]), 58.1% indicated a time interval between 1 and 2 h, while a further 2.6% believed that a 4-year-old child can spend more than 2 h in front of a screen.

### 3.2. Differentiating Factors of Parental Health Literacy 

When exploring the differentiating factors of parental health literacy, we included the parents’ highest education, healthcare qualification, and the presence of any chronic illness in the child as independent variables in the analysis (Table 4). 

Regarding the highest level of education, there was no significant difference between the groups (χ^2^ = 4.970; df = 4; *p* = 0.290), however, we found that among parents with sufficient health literacy, those with a higher education level were represented in the highest proportion. In terms of educational background, we also found it important to ask whether the respondent had a qualification in some field of healthcare, since obviously, this type of training conveys knowledge that provides the participants with more accurate information about health and illness. Not surprisingly, those who received this type of education have significantly higher health literacy, regardless of the level of training (χ^2^ = 6.400; df = 2; *p* = 0.041). A total of 7.2% (*n* = 43) of the responding parents raised a child with a chronic disease, through whom they regularly came into contact with the healthcare system due to treatments and examinations. In our study, however, we did not find a significant correlation between raising a child with a chronic disease and parental health literacy (χ^2^ = 5.554; df = 2; *p* = 0.062).

### 3.3. Differentiating Factors of Health-Promoting Habits and Daily Routines among Kindergarten Children

Some of the habits we examined can be observed in young children very early, even before the age of three, while other habits become routine actions at later ages, which is why we carried out our analyses by age groups. In Table 5, we present the cumulative values of the “often” and “always” responses by age group.

Analyzing the data in Table 5, it can be observed that the older the child, the more typical it is that the examined habit or daily routine is integrated into the child’s daily activities and habit system. Regarding eating, significant differences can be observed primarily in the field of independence between individual age groups, and in relation to activities that are associated with the development of fine motor and coordination skills, such as using a knife. In terms of hygiene habits, no significant differences were found between the examined age groups in the case of habits related to toilet use; at the same time, we identified significant differences regarding blowing one’s nose independently in a tissue, thorough toothbrushing, and behavior when coughing and sneezing. In terms of gender differences, we found significant differences in nine habits using Fisher’s Exact Test; these are shown in Table 6. The results show that the habit system of girls is more developed than that of boys in several areas. This is the most evident in hygiene habits (handwashing, nose blowing, and behavior when coughing and sneezing), but also in habits related to eating, dressing, and water conservation. Furthermore, it is important to point out that verbal conflict resolution is also more characteristic of girls.

### 3.4. The Correlations between Parental Health Literacy and the Habit System

For our research, the most important question to answer was whether parental health literacy shows a correlation with the habits observed in children, and if so, in which areas. In Table 7, we list the habits and activities related to daily routines, where, with one exception, a significant difference can be verified between the groups of parents with different levels of health literacy. In the table, we merely indicate how frequently certain habits or routine actions are often or always observed among the children of parents with sufficient health literacy. 

Based on our results, it can be concluded that hygiene habits are a significantly more frequent part of the daily routine for children of parents with higher health literacy. The exception to this is handwashing after toilet use without being asked, where we could not prove a significant difference between the groups having different levels of health literacy; however, it is characteristic of the children of parents with higher health literacy in a greater proportion, and also fits into the above-mentioned correlation that the existence of hygiene-related habits is more typical for children of parents with higher health literacy. In addition to hygiene habits, it is important to point out that in the case of habits that require greater cooperation, consistency, and awareness, the indicators are more favorable for parents with higher health literacy: the evening bedtime, the adherence to safety rules related to accident prevention, the restrictions of media tools, and the choice of activities that require high-intensity physical activity are integrated to a greater extent into the children’s daily routine. 

The correlation between parental education and children’s daily habits proved to be significant in several areas (Table 8). In general, it can be said that the children of more educated parents are ahead of their peers in terms of habit formation, especially in the case of habits that require a higher degree of cooperation and awareness. This includes, among other things, accepting the restrictions on the consumption of sweets and the evening bedtime set by the parent, following safety rules, and prioritizing physical activity over screen time and other sedentary activities.

Our results show that regarding the parents’ education, the most important differentiating factor is the existence of a secondary school-leaving exam, meaning an upper secondary level of education; so, compared to those with primary and lower secondary education, the indicators for children of parents with a school-leaving certificate or a degree are much more favorable. 

The analysis of factors influencing children’s health-promoting habits revealed that parental education level, parental health literacy level, and the gender and age of the child are relevant as explanatory variables; therefore, these variables were included in the logistic regression analysis. In the first step, we examined those habits where, in addition to parental health literacy, another variable resulted in significant differences in terms of health-promoting habits (Table 9). Regarding the habits included in the logistic regression analysis, we found that the significant effect of parental health literacy remained with the exception of one habit (20. They understand and accept the evening bedtime I set). 

After that, we included further statements in the analysis where, in addition to parental health literacy, two other explanatory variables showed a significant correlation with the existence of health-promoting habits. The health-promoting habits included in the analysis were the following: (a) 14. They are able to blow their nose into a tissue on their own; (b) 15. They put their hand in front of their mouth when sneezing or coughing; and (c) 22. They follow safety rules during their play activities. In the case of all three habits, our analyses pointed to the more pronounced differentiating effect of the variable “child’s age”, which entered the model first, while the significant effect of “parental health literacy” disappeared.

## 4. Discussion

The education of young children is primarily based on habitual actions, in the development of which the example provided by the parents and educators and their supportive and affirming presence have an irreplaceable role. Habits can be formed most effectively up to the age of nine [37], which also designates the relevant institutions; in addition to the family, nursery schools, kindergartens and the lower grades of primary school are decisive in habit formation [57]. 

In relation to the advancement of children’s habit systems, we did not identify significant age differences in the case of all habits, and this was especially true for some hygiene habits. There were no differences in handwashing by age group, which we believe can be explained by the increased attention paid to the role of handwashing in both family and institutional education in recent years due to the COVID-19 pandemic. Additionally, no significant difference was found in terms of toilet usage habits; since the majority of children are completely toilet trained by the time they start kindergarten, toilet usage routines are already part of their everyday lives. At the same time, in the case of some hygiene-related habits, there were significant differences between the children; since learning the correct technique of both blowing the nose and brushing the teeth requires a lot of practice, children are typically able to perform these techniques well only at the age of five or later. The use of a hand or a handkerchief in front of the mouth when coughing or sneezing requires continuous awareness from the children, the development of which is a time-consuming process.

During the foundation of health-supporting habits in early childhood, special attention should also be paid to accident prevention. In this regard, the effectiveness of health education increases if we can make the young child understand the rules of accident prevention. Although at first glance, the statement (23) “They safely avoid products that are dangerous for them (e.g., medicines, chemicals, alcohol) and poisonous plants” indicates particularly favorable health behavior in the case of children under four years, we must note that in this respect children under the age of four have little autonomy. On the one hand, the adults around them remove dangerous products from the children’s reach, and on the other hand, because of constant supervision, the children may not even have the opportunity to come into contact with dangerous products. Regular compliance with safety rules during play activities can be observed in the highest proportion among 5–6 year olds. In the case of children older than this, probably due to their age, the elements of play are likely to be more dangerous in themselves.

In addition to age differences, based on the answers of preschool children’s parents, gender differences also emerged; in several habit behaviours, girls are ahead of boys. We believe that the background of all this is mainly the different socialization of boys and girls. Girls are facing higher expectations to follow rules and boys are being less punished by the environment for nonconforming behavior (fighting, ignoring rules) [58].

In our study, we examined the habit systems of preschool-age children in relation to the level of their parents’ health literacy. Understanding health literacy is of particular importance in identifying the areas where knowledge transmission, attitude shaping, and the reinforcement of health-promoting habits can ensure long-term health preservation. To assess health literacy, we used the HLS-EU-Q16 questionnaire containing 16 statements. According to our data, 13.4% of the respondents belong to the group of parents with inadequate health literacy, which is almost the same (13%) as what was previously measured among Hungarian parents (mothers). Similar to the study of Sántha (2021) [9], the parents involved in our study also find it difficult to judge the reliability of information from the media. Overall, our results on the health literacy of parents show that the health literacy of the sample is higher than that of the Hungarian population as a whole [59]. This is probably due to the fact that during the period of childbearing and having a young child, young parents are more informed about childcare, the preservation of the child’s health, and the treatment of diseases, and during this period they more often meet health professionals owing to vaccinations, regular screening tests, and childhood diseases. 

In addition to using the HLS-EU-Q16 questionnaire, we collected information on whether parents involved in the study were aware of international recommendations related to certain early childhood routines. While the majority of parents gave answers in accordance with international recommendations on the time of the first toothbrushing and the recommended duration of sleep and rest, many of them have incorrect information about physical activity and screen time. This also suggests that although the data of the standardized questionnaire show a higher level of health literacy among the parents, in the case of specific questions, there is still a significant lack of information among them.

Regarding the parents’ health literacy, only health qualification proved to be a differentiating factor. We did not observe any differences in terms of education or settlement type of residence, and there was also no correlation between the age of the parents and the level of health literacy. Previous research [9,11,12] confirmed a stronger differentiating effect of sociodemographic characteristics. 

In our study, the exploration of parental health literacy was primarily important for us in connection with young children’s habit systems. In Hungary, the family and the kindergarten cooperate closely in the formation of children’s habits. Our analyses confirm that by the time children enter kindergarten, some of the basic hygiene habits and daily routines have already been established, while many of the habits that require longer practice and a higher level of awareness are integrated into the children’s habit system only by the end of kindergarten.

In addition to the differences resulting from the age characteristics and the gender of the children, we found several correlations that point out that the parents’ higher health literacy and education have a positive effect on the formation of preschool children’s habit systems. A higher proportion of parents with at least sufficient health literacy reported that basic hygiene habits and daily routines, as well as habits related to safety, accident prevention, and restrictions of media tools, were already integrated into their children’s habit system.

For the children of parents with at least upper secondary education, the interiorization of habits related to daily routines and of the ones requiring greater insight and cooperation is realized at a higher level. As a result of this, when children of more educated parents start primary school, they are in a more advantageous position, not only in terms of cultural capital [60,61]. Since habits have a mind-freeing role, well-established habits that form a system no longer require a conscious effort from children; children do not have to concentrate on practiced actions, so they have more capacity to learn other things. The acquisition of a habit system provides a sense of security and, at the same time, confidence, which greatly facilitates institutional socialization and allows children to focus on their learning tasks. 

The multivariate analyses showed that each of the factors filtered out during the bivariate analyses supports children’s health-promoting habits. In most cases, parental health literacy, which proved to be a differentiating factor, maintained its significant effect on the majority of health-promoting habits included in the multivariate analysis, even after controlling for the child’s age and gender and the parental education level.

The main limitations of the study relate to the applied method and representativeness of the sample. The data of the questionnaire survey are based on self-reports, so the effort to meet expectations may influence responses. Since the respondents commented on their own children’s habits, parental bias cannot be ruled out. Although the number of respondents was relatively high, we cannot draw general conclusions due to the characteristics of sampling (convenience sampling).

## 5. Conclusions

Our study was aimed at exploring the correlations between the health-promoting habits of preschool-aged children and the health literacy of their parents. Our results show that, in addition to parental health literacy, children’s health-promoting habits are fundamentally influenced by the child’s gender and age, as well as their parent’s education level. The high level of parental health literacy is associated with the advancement of the child’s habit system; therefore, the enhancement of parental health literacy may contribute to the child’s smooth socialization and healthy lifestyle even in the case of a lower parental education level.

Understanding the habit system of preschool children can become comprehensive through the involvement of both parents and professionals working in early childhood education. In order to explore this issue more thoroughly, it is essential to develop a measurement tool that covers all elements of health-promoting habits in early childhood, emphasizing the age-specific characteristics. The reliability of the results can be increased by systematic observation of children during daily routine activities.

In the long term, habit formation in early childhood can establish the interiorization of positive health behavior motives for the future, which are important components of a healthy lifestyle and contribute to the emergence of favorable physical, mental, and social health in adulthood.

## Figures and Tables

**Table 1:** Reliability of applied scales.

Scale	Number of Items	Mean ± SD	Cronbach’s Alpha
Healthcare (sub-index)	7	36.05 ± 8.33	0.82
Disease prevention (sub-index)	5	35.99 ± 8.68	0.71
Health promotion (sub-index)	4	36.93 ± 9.26	0.78
Overall health literacy index	16	36.21 ± 7.34	0.89

**Table 2:** Respondents’ sociodemographic characteristics (*n* = 598).

	*n*	%
Gender		
Men	85	14.2%
Women	513	85.8%
Education level		
Lower secondary education or less	173	29.0%
Upper secondary education or post-secondary education	211	35.3%
Tertiary education (BA, MA, PhD)	214	35.8%
Healthcare or medical qualification		
Yes	63	10.5%
No	535	89.5%

**Table 3:** Distribution of answers to the items of the HLS-EU-Q16 scale (%) *n* = 598.

How Easy or Difficult Is It to…	Very Difficult/ Difficult		Easy/ Very Easy		Don’t Know/ No Answer	
	*n*	%	*n*	%	*n*	%
Healthcare system-related competence sub-index						
1. Find information on treatment of illnesses that concern you?	109	18.2	463	77.4	26	4.3
2. Find out where to get professional help when you are ill?	157	26.3	430	71.9	11	1.8
3. Understand what your doctor tells you?	92	15.5	499	83.8	5	0.8
4. Understand your doctor or pharmacist’s instructions on how to take a prescribed medicine?	16	2.6	579	97.0	2	0.3
5. Judge when you may need to get a second opinion from another doctor?	223	37.4	334	56.0	40	6.7
6. Use information the doctor gives you to make decisions about your illness?	137	22.9	442	73.9	17	2.8
7. Follow instructions from your doctor or pharmacist?	22	3.7	572	95.8	3	0.5
Prevention sub-index						
8. Find information on how to manage mental health problems like stress and depression?	146	43.8	405	67.8	47	7.9
9. Understand health warnings about behavior such as smoking, low physical activity and drinking too much?	26	4.4	558	93.3	14	2.3
10. Understand why you need health screenings?	13	2.2	578	97.0	5	0.8
11. Judge if the information on health risks in the media is reliable?	228	38.2	328	55.1	40	6.7
12. Decide how you can protect yourself from illness based on information in the media?	245	41.0	316	52.8	37	6.2
Health promotion sub-index						
13. Find out about activities that are good for your mental well-being?	91	15.2	480	80.3	27	4.5
14. Understand advice on health from family members or friends?	61	10.2	525	87.8	12	2.0
15. Understand information in the media on how to become healthier?	133	22.2	437	73.0	28	4.7
16. Judge which everyday behavior is related to your health?	61	10.2	524	87.7	12	2.0

**Table 4:** Differentiating factors of parental health literacy.

	Inadequate HL	Problematic HL	Sufficient HL	χ^2^	*p*-Value
Education level					
Lower secondary education or less	16.2%	28.3%	55.5%	4.970	0.290
Upper secondary education or post-secondary education	15.2%	30.3%	54.5%		
Lower secondary education or less	9.3%	32.7%	57.9%		
Healthcare or medical qualification					
Yes	12.7%	17.5%	69.8%	6.400	0.041 ^1^
No	13.5%	32.1%	54.4%		
Raising child with chronic disease					
Yes	13.5%	29.4%	57.1%	5.554	0.062
No	11.6%	46.5%	41.9%		

^1^ Effect size ≤ 0.2 measured by Cramer’s V.

**Table 5:** Distribution of regularly performed healthy habits and daily routines by age.

They…	Under 4 Years	4–5 Years Old	5–6 Years Old	Over 6 Years	χ^2^	*p*-Value
1. eat independently, without help.	91.7%	97.5%	99.5%	99.1%	17.626	0.001 ^1^
2. use the spoon and the glass properly when eating.	96.9%	97.0%	98.5%	100%	4.103	0.251
3. use the fork properly when eating.	95.8%	92.4%	98.5%	97.2%	9.649	0.022 ^1^
4. know and practice the proper use of the knife in connection with meals.	51.0%	58.6%	69.9%	82.4%	28.361	<0.001 ^2^
5. are capable of self-service at meals, pouring and scooping independently, according to their individual needs.	59.4%	68.0%	84.2%	85.2%	32.446	<0.001 ^2^
6. like to eat a variety of fruit and vegetables.	82.3%	83.8%	82.7	75.9%	3.163	0.367
7. understand and accept restrictions on the consumption of sweets.	67.7%	70.2%	75.0%	72.2%	2.040	0.564
8. eat in a civilized manner (mouth closed, use of napkin).	91.7%	93.9%	95.9%	96.3%	3.056	0.383
9. wash their hands before meals without being asked.	74.0%	73.7%	80.1%	75.9%	2.581	0.461
10. know and apply the correct handwashing technique.	93.8%	92.45	95.4%	90.7%	2.761	0.430
11. use water sparingly.	62.5%	61.6%	76.5%	72.2%	12.514	0.006 ^1^
12. wash their hands after using the toilet without being asked.	85.4%	81.8%	87.8%	90.7%	5.404	0.144
13. flush the toilet and lower the lid after use.	87.5%	88.4%	89.2%	90.7%	0.600	0.896
14. are able to blow their nose into a tissue on their own.	75.0%	85.4%	94.9%	94.4%	30.660	<0.001 ^2^
15. put their hand in front of their mouth when sneezing or coughing.	82.3%	82.8%	90.8%	93.5%	11.688	0.009 ^1^
16. are cooperative during bathroom activities at home (bathing, hair washing).	95.8%	92.4%	97.4%	96.3%	5.998	0.112
17. brush their teeth independently and thoroughly.	82.2%	83.8%	93.4%	91.7%	12.280	0.006 ^1^
18. when dressing, show independence and sophistication appropriate to their age.	90.6%	90.9%	94.45	94.4%	2.836	0.418
19. go to bed around the same time every night.	93.8%	93.4%	94.9%	93.5%	0.427	0.935
20. understand and accept the evening bedtime I set.	88.5%	89.4%	91.8%	88.9%	1.147	0.766
21. wake up well rested, even on weekdays.	93.8%	86.4%	90.8%	89.8%	4.328	0.228
22. follow safety rules during their play activities.	84.4%	86.4%	96.4%	88.0%	15.197	0.002 ^1^
23. safely avoid products that are dangerous for them (e.g., medicines, chemicals, alcohol) and poisonous plants.	99.0%	94.9%	98.5%	100%	10.218	0.017 ^1^
24. are cooperative in the use of safety devices during transport (e.g., crash helmet, safety seat and belt).	93.8%	94.9%	96.9%	94.9%	1.927	0.588
25. understand and accept restrictions on the use of media tools.	76.0%	80.8%	81.6%	81.5%	1.449	0.694
26. prefer virtual games (tablet, phone, computer, etc.) rather than games that require movement.	24.0%	26.3%	25.0%	28.7%	0.727	0.867
27. prefer indoor games that require little movement to outdoor activities.	20.8%	22.7%	17.9%	26.9%	3.565	0.312
28. like to participate in activities involving intense movement (e.g., ball games, tag, etc.)	95.8%	93.9%	94.9%	89.9%	4.052	0.256
29. express their feelings verbally in case of conflict, tension, or anxiety.	84.4%	83.2%	86.2%	92.6%	5.383	0.146
30. can play intensely, even without the support of an adult, for at least half an hour.	82.3%	86.9%	93.9%	90.7%	10.528	0.015 ^1^

^1^ Effect size ≤ 0.2 measured by Cramer’s V; ^2^ Effect size: between 0.21 and 0.3 measured by Cramer’s V.

**Table 6:** Distribution of regularly performed healthy habits and daily routines by gender (%).

They…	Boy	Girl	*p*-Value
6. like to eat a variety of fruit and vegetables.	77.5%	85.1%	0.024
9. wash their hands before meals without being asked.	72.1%	80.2%	0.030
10. know and apply the correct handwashing technique.	90.7%	95.5%	0.030
11. use water sparingly.	63.9%	72.2%	0.038
12. wash their hands after using the toilet without being asked.	80.7%	90.3%	0.001
14. are able to blow their nose into a tissue on their own.	83.9%	92.7%	0.002
15. put their hand in front of their mouth when sneezing or coughing.	84.3%	90.6%	0.030
18. when dressing, show independence and sophistication appropriate to their age.	89.6%	95.5%	0.010
29. express their feelings verbally in case of conflict, tension, or anxiety.	81.0%	91.7%	<0.001

Effect size ≤ 0.2 measured by Phi (in case of all items).

**Table 7:** Healthy habits and daily routines regularly performed by children of parents with sufficient health literacy.

They…	Parents with Sufficient Health Literacy	χ^2^	*p*
9. wash their hands before meals without being asked.	80.0%	6.377	0.041
12. wash their hands after using the toilet without being asked.	88.7%	5.211	0.074
14. are able to blow their nose into a tissue on their own.	90.4%	6.130	0.047
15. put their hand in front of their mouth when sneezing or coughing.	90.1%	6.310	0.043
16. are cooperative during bathroom activities at home (bathing, hair washing).	97.6%	9.456	0.009
17. brush their teeth independently and thoroughly.	91.9%	9.714	0.008
19. go to bed around the same time every night.	96.7%	11.010	0.004
20. understand and accept the evening bedtime I set.	92.5%	6.057	0.048
22. follow safety rules during their play activities.	92.2%	10.498	0.005
23. safely avoid products that are dangerous for them (e.g., medicines, chemicals, alcohol) and poisonous plants.	99.4%	10.467	0.005
24. are cooperative in the use of safety devices during transport (e.g., crash helmet, safety seat and belt).	97.6%	9.321	0.009
25. understand and accept restrictions on the use of media tools.	85.4%	11.815	0.003
28. like to participate in activities involving intense movement (e.g., ball games, tag, etc.)	96.7%	11.067	0.004

Effect size ≤ 0.2 measured by Cramer’s V (in case of all items).

**Table 8:** Correlations between children’s habits and parental education level.

They…	lower Secondary/ Less	Upper Secondary/ Post-Secondary	Tertiary (Graduates)	χ^2^	*p*
7. understand and accept restrictions on the consumption of sweets.	64.2%	73.9%	75.7%	7.057	0.029 ^1^
8. eat in a civilized manner (mouth closed, use of napkin).	92.5%	93.4%	97.7%	6.070	0.048 ^1^
16. are cooperative during bathroom activities at home (bathing, hair washing).	90.8%	97.2%	97.2%	11.373	0.003 ^1^
18. when dressing, show independence and sophistication appropriate to their age.	86.1%	94.8%	95.8%	15.316	<0.001 ^1^
19. go to bed around the same time every night.	86.7%	96.2%	97.7%	23.077	<0.001 ^2^
20. understand and accept the evening bedtime I set.	82.7%	94.3%	91.5%	15.218	<0.001 ^1^
22. follow safety rules during their play activities.	85.0%	88.6%	94.4%	9.493	0.009 ^1^
26. prefer virtual games (tablet, phone, computer, etc.) rather than games that require movement.	36.4%	26.5%	16.8%	19.193	<0.001 ^1^
27. prefer indoor games that require little movement to outdoor activities.	29.5%	20.4%	16.4%	10.014	0.007 ^1^
28. like to participate in activities involving intense movement (e.g., ball games, tag, etc.)	89.0%	95.3%	96.3%	9.827	0.007 ^1^

^1^ Effect size ≤ 0.2 measured by Cramer’s V; ^2^ Effect Size: between 0.21 and 0.3 measured by Cramer’s V.

**Table 9:** Association of parents’ health literacy and their children’s health-promoting habits according to logistic regression analyses.

They…	β	SE	Wald	*p*-Value	Exp (B)
9. wash their hands before meals without being asked.	0.308	0.133	5.33	0.021	1.361
16. are cooperative during bathroom activities at home (bathing, hair washing).	0.540	0.245	4.847	0.028	1.716
17. brush their teeth independently and thoroughly.	0.479	0.167	8.286	0.004	1.615
19. go to bed around the same time every night.	0.492	0.221	4.953	0.026	1.635
20. understand and accept the evening bedtime I set.	n.s. *				
23. safely avoid products that are dangerous for them (e.g., medicines, chemicals, alcohol) and poisonous plants.	0.854	0.342	6.253	0.012	2.350
28. like to participate in activities involving intense movement (e.g., ball games, tag, etc.)	0.602	0.216	7.775	0.005	1.826

* n.s. no significant difference

## Data Availability

The data presented in this study are available on request from the corresponding author due to protect the data privacy and restrict unauthorized use.
